# Annotations

**Published:** 1896-05-09

**Authors:** 


### Annotations.

### Meat and Milk from Sewage Farms.

A medical contemporary raises the question of the injuriousness or otherwise to health of meat and milk produced on sewage farms. This is a question which cannot be settled with a precision approaching to the mathematical, either in physiological laboratories or by means of statistics gathered from the experience of the community at large. But there is an old scientific method, now a good deal despised, whereby men of intelligence can arrive at trustworthy conclusions; and that is the method of careful observation, personal experiment, and an appeal to the court of common sense. By the use of this method many facts like the following have been accumulated by generations of experimentalists, and they settle the controversy in a practical sense. For example, if a cow is fed on turnips, within twenty-four hours her milk will taste of turnips, and if butter be churned from the cream, the butter will taste too. The intensity of the turnip flavour is the measure of the quantity of turnips taken. In like manner, if pigs be fed on horseflesh, as they often are, their bacon will taste of the horseflesh; if they be fed on fish, the bacon has a fishy taste. The same is true of hens and their eggs. Feed hens on decaying animal matter, which they will eat greedily, and both their eggs and flesh will be most unpleasant and unwholesome eating. In the case of ducks the facts are much more striking. Ducks are very unclean feeders. Give them abundance of garbage, and they will refuse corn and similar food. Their flesh is then most pungent to the taste, and in many people is so potently poisoning as to produce diarrhœa. Animals fed on sewage farms under certain conditions are liable to have their flesh and secretions changed in character by the sewage-produced herbs and grasses upon which they feed. If the sewage on a given farm be so managed that no more of it be put into the soil than any given crop can adequately deal with, then the crop will be sweet and natural, and the cattle or other animals fed on it will be sweet and natural too. But if the soil be gorged to repletion with sewage, then the crops will be surcharged with sewage elements, and unfit for food, and the meat and milk of animals fed on such crops will be like the crops, and very unpleasant to the taste as well as dangerous to health. It is in the last resort all a question of the intelligence and conscience of the managers of sewage farms.

### Post mortems on Hospital Patients.

The remarks made by Mr. Braxton Hicks at a recent inquest at St. Thomas's Hospital in regard to post-mortem examinations are worthy of consideration by all house surgeons. What is the exact legal status of hospital authorities as to post-mortems is a matter in regard to which there may be some question. Probably they have no rights, while, on the other hand, the friends have no remedy when the thing has been done. But, so far as concerns accidents, and all cases which the coroner has to investigate, the law is clear. Certain places are appointed to which such a body may be carried, after which no one is allowed to touch the corpse except by direction of the coroner. Mr. Braxton Hicks raised objection to two practices, both of which are common enough at other hospitals besides St. Thomas's. One was the routine making of post-mortems without his permission, the other the fact that the individual who gave evidence at the inquest had not always been present at the post-mortem, and thus could but read notes taken by someone else—which was not evidence. He pointed out that it might at times be desirable, in the interests of justice, that the post-mortem should be made by some other individual than the one to whom the duty would naturally fall, and that, in any case, the coroner had the right both to decide whether or no the autopsy should be made and to say by whom it should be performed. So long as hospital authorities show proper deference to the position of the coroner, we do not think that any difficulty need often be raised on this point, although undoubtedly the coroner possesses very large powers in the matter. But, as regards the other point, the hospitals certainly should make such arrangements as to ensure that the house surgeon who sees the case on admission shall also witness the post-mortem, and thus be able to give evidence at first hand as to the whole of the case. If this is not done, the coroner is clearly within his rights in issuing a subpœna for the attendance of the pathologist as well as of the house surgeon.

### Rabbits—The Titbits of the Poor.

The London poor are exceedingly fond of rabbits, or at least one may judge so by the fact that such extraordinary numbers are sold in the poorer parts of London every week. The rabbit, even if he sometimes happens to be a carnivore instead of a rodent, is very nice eating, provided always that he is young, plump, and entirely fresh. When he is not fresh, he is an abomination, and a most deadly one. To the poor the titbit at dinner or supper is a real pleasure; and a real pleasure in the lot of those whose pleasures are necessarily few ought not to be allowed, without protest, to be turned into a danger to health. Yet that is what is now happening, if we may trust the recently issued report of the medical officer of health for the port of London. Rabbits, the medical officer tells us, are imported into London in very large numbers. A considerable proportion come from Australia; and those, contrary to expectation, mostly arrive sweet and in excellent condition, although they travel as much as ten or twelve thousand miles. On the other hand, the Ostend rabbit, although he comes but from the other side of the Channel, often arrives here in a condition of deadly putridity, and indeed he is not seldom quite putrid before he leaves the town of export. What one feels is, that the poor man's titbits should be guarded for him by a much more competent system of inspection; but what one feels very much more is that there are tens of thousands of acres in England only fit for use as rabbit warrens, and that if we had but the energy to develop these, the poor man would get his titbits in a perfectly wholesome condition, and the poor man's money would be exceedingly useful to the farmer or the landed proprietor who might be responsible for the production and marketing of the rabbits.

